# To the Editors of the Medical and Physical Journal

**Published:** 1807-06

**Authors:** William Nosworthy

**Affiliations:** Crediton, Devonshire


### *To the Editors of the Medical and Physical Journal.*

Gentlemen,

INCLOSED, I send you the drawing of a Fœtus, which a poor Woman in this neighbourhood was delivered of, about a fortnight since. I was called to her, when the pains had already effected the delivery; and, on examining the child, to whlch my attention was directed by the strong accounts of the attendants, the appearances were so singular, that I requested permission to examine it at my leisure. It had been dead some time, for the epidermus was loose, but no mortification had taken place. Though the woman had nearly completed her full time, it was but of about half the usual size. The vertex and occiput were wanting, a flattish surface extending obliquely downwards, from a little above the eyes to the neck, covered with a membrane darker than the skin, resembling somewhat the appearance of a cicatrix. Under this oblique surface, no brain was discoverable, but the whole was a bony mass. From the deficiency of forehead, the eyes appear prominent; the nose is flat; the ears large and high; the neck short, and the under jaw-bone divided, which may have, however, happened in conveying it to my house. At the back part of the neck is a small hole; through which a probe will pass to the bottom of the vertebral cavity; so that the child seems destitute of brain and spinal marrow; or at least to possess a small proportion of either. The right arm is considerably shorter than the other, and the hand bent upward, at the wrist, so as to resemble a foot, and it terminates in four fingers only. About the middle of the arm is a joint, without natural curvature, at which the limb can be bent in any direction. *(Vide plate.)*

I am, &c.

WILLIAM NOSWORTHY.

*Crediton, Devonshire,*
*Feb.* 18, 1807.

---

